# Variations in Dissolved Nitrate, Chloride, and Sulfate in Precipitation, Reservoir, and Tap Waters, Columbus, Ohio

**DOI:** 10.3390/ijerph15081752

**Published:** 2018-08-15

**Authors:** Deborah L. Leslie, W. Berry Lyons

**Affiliations:** 1 Department of Physical Sciences, Arkansas Tech University, Russellville, AR 72801, USA; 2 School of Earth Sciences and Byrd Polar and Climate Research Center, The Ohio State University, Columbus, OH 43210, USA; lyons.142@osu.edu

**Keywords:** water supply, anion chemistry, Ohio precipitation, urban reservoirs

## Abstract

Urban potable waters can be very susceptible to human activities that can impact water quality and, hence, public health. Columbus, Ohio, is currently the 14th largest city in the United States with an estimated population of ~860,000. Much of the urban population receives its water supply from a series of reservoirs located north of the city proper. These reservoirs are fed by river systems that drain either large agricultural lands, or rapidly growing suburban areas, or both. The agricultural activities introduce dissolved nitrate, and increased usage of de-icing salts on roads and highways within the drainage area introduce chloride into the river/reservoir systems. High nitrate in drinking water poses a potential health risk, particularly to infants, while high chloride, applied as halite, in drinking water can aid in the development of cardiovascular disease. In this work, we present a 19-month time series measuring nitrate, chloride, and sulfate in local precipitation, reservoir and household tap waters in order to better understand the relationship of the hydrologic residence time on the tap water chemistry, as well as to evaluate the anion concentrations. The highest chloride tap water concentration, 6.9 mM, occurred in early February 2011, while increases in nitrate occurred in both early summer and the middle of winter. In general, the anion concentrations in the precipitation are all equal to or lower than the reservoir waters. Similarly, the tap water had concentrations of chloride and sulfate higher than reservoir water, while nitrate was similar to reservoir water. Tap water had higher fluoride and sulfate concentrations, suggesting that they are added during the treatment of the reservoir water prior to residential distribution. These data clearly demonstrate the importance of watershed lands on the quality of water in the human distribution system.

## 1. Introduction

One of the top priorities for urban development, sustainability, and the protection of public health is the provision of safe drinking water [1]. The quality of water for urban consumption is based in large part on its initial quality, the storage and treatment of the water supply, as well as the interaction of the water with the distribution system. The greatest contributor to many water supplies in urbanized areas is surface water. The initial water quality is a function of the chemistry of the precipitation and the physical-chemical interactions of the precipitation and the watershed materials through which it flows. The overall management of urban water supplies, from watershed to tap, can have a profound impact on human health. Therefore, the evaluation of water resource management to provide potable water is essential for the sustainability of water supplies.

The primary anions in most precipitation are chloride, sulfate, and nitrate. Numerous anthropogenic activities can influence the initial water quality, thus increasing these anions in human water supplies. For example, chloride is a ubiquitous water pollutant, coming from a variety of potential sources [2]. However, recent work in the U.S. reports that areas receiving frozen precipitation strongly indicates that road de-icing salt is a major contributor, and its source has increased dramatically over the past few decades [3,4]. This is also the case in Ohio, where temporal trends indicate increases of chloride in a number of rivers since the 1960s that have been attributed to road salt [5]. Although chloride is non-toxic, concentrations >7.0 mM in water are of concern to human health [3].

The World Health Organization (WHO) has no health based guideline for sulfate in drinking water. Because of its potential laxative effects, sulfate has been suggested as a possible cause of diarrhea, especially in children. However, there has been no clear association between elevated sulfate in drinking water and the occurrence of infant diarrhea [6], but there have been few experimental studies on the topic [7].

Nitrate is a major groundwater and surface water pollutant in the U.S. [8]. It can have numerous sources, which include fossil fuel burning, human and animal waste products, and agricultural runoff from fertilizer use. Nitrate is regulated in human drinking water at 10 mg L^−1^ or ~0.7 mM [9]. There is controversy regarding the effect, if any, on the consumption of high nitrate water on other diseases, such as bladder and thyroid cancer. The major hazard of high nitrate in water is methemoglobinemia or “blue-baby” syndrome. In this disease, nitrate is converted to nitrite in the intestinal tract and that in turn oxidizes the ferrous iron in hemoglobin to ferric iron, thereby minimizing oxygen uptake [10]. It is rare in adults, as most victims are infants of less than six months [10]. The testing of public water supplies is extremely important in minimizing this disease. Public water supplies that either drain (surface water) and/or underlie (groundwater) highly agricultural landscapes must maintain monitoring, because the potential of water high in nitrate continues to exist. This is particularly true in the U.S. Corn Belt region where the use of fertilizer for corn agricultural and the planting of N_2_-fixing legumes, such as soya, have produced nitrate-enriched water [11].

It is essential to understand the relationship between water resource availability and climatological conditions for developing strategies to ensure long-term sustainability of water supplies [12]. With increasing suburbanization in nearby urban areas of Columbus, Ohio, the need to understand current water resource dynamics is extremely important in terms of anticipation of future meteorological and hydrological conditions. Our previous work has demonstrated that temporal meteorological variations over monthly to multiple monthly periods greatly impacted the residence time of water in the watersheds that flow into the reservoirs [13]. The purpose of this work was to evaluate the relationship between the geochemistry of local precipitation, reservoir waters derived from the drainage from agricultural lands north of the city of Columbus, and household tap water over 19 months, during much of that time with above average precipitation amounts. We investigated the chemical connection between tap water, reservoir water and precipitation under the premise of the importance of watershed lands on the quality of water in the human distribution system. This work concluded that the major source of nitrate to the reservoir-tap water continuum is from anthropogenic addition upstream from the reservoirs themselves.

## 2. Materials and Methods

### 2.1. Columbus, Ohio, Water Supply

In 2016, Columbus, Ohio, was the 14th largest city in the United States with an approximate population of 860,090 [14]. A secure municipal water supply played a crucial role in the population growth of Columbus over the past 100 years. Columbus has increased its water supply storage by the use of three reservoirs, Griggs, O’Shaughnessy, and Hoover, which provide 85% of the more than 4.9 × 10^5^ m^3^ of daily water supplied to the metropolitan area. The remaining 15% is drawn from wells in southern Franklin County, Ohio [15]. More recently in 1978, the Alum Creek Reservoir was built as a supplement supply to the Hoover Reservoir. Leslie et al. [13] provides the complete water supply history and describes each reservoir capacity for storage and demand.

### 2.2. Site Description—Reservoirs and Water Supply Distribution

**Figure 1:**
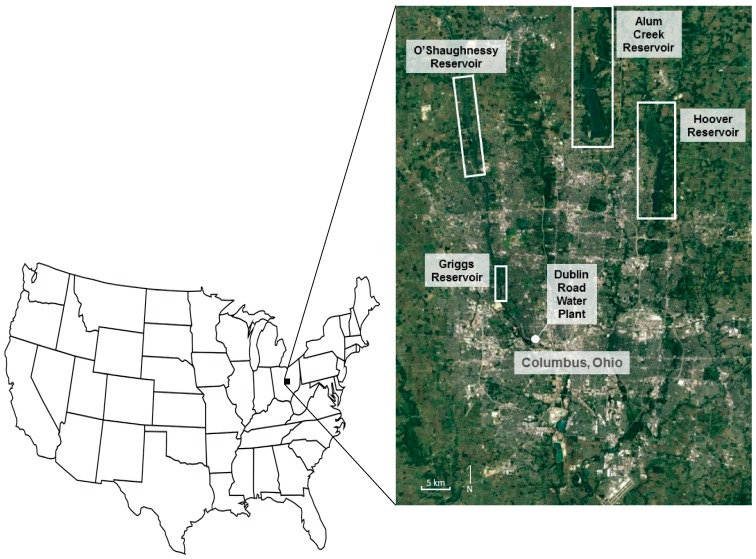
The reservoirs that contribute to the Columbus, Ohio, water supply are Griggs, O’Shaughnessy, Alum Creek, and Hoover [13]. The Dublin Road Water Plant extracts water from Griggs and O’Shaughnessy for water supply usage.

Griggs Reservoir (Figure 1), located on the northwest side of Columbus, Ohio, is ~9.7 km long and associated with the Scioto River. Approximately 16 km upstream of Griggs Dam on the Scioto River is the O’Shaughnessy Dam, separating the slightly larger O’Shaughnessy Reservoir (Figure 1) from Griggs Reservoir. Together, these two reservoirs (Griggs and O’Shaughnessy) have a capacity of 2.3 × 10^7^ m^3^ [15]. Approximately 30% of the drinking water for the city of Columbus comes from the Griggs and O’Shaughnessy Reservoirs. The Griggs and O’Shaughnessy Reservoirs are more of a riverine system connected to the Scioto River, which creates a very weather- and flow-dependent system [16]. The Griggs and O’Shaughnessy Reservoirs hold only a small percentage of the Scioto River water that flows through Columbus. The average residence time of water in the O’Shaughnessy Reservoir is approximately 26 days [16]. The Dublin Road Water Plant (DRWP; southwest section in Figure 1) utilizes surface water from the Griggs and O’Shaughnessy Reservoirs on the Scioto River, and provides water to downtown Columbus, western, and southwestern Franklin County. The DRWP is designed to treat up to 2.5 × 10^5^ m^3^ day^−1^. Leslie et al. [13] reported a residence time of approximately 60 days for water movement from the reservoirs to a DRWP household tap.

Hoover Dam (Figure 1) forms the Hoover Memorial Reservoir, and it supplies water for the entire northeast portion of Franklin County. The residence time of water in the Hoover Reservoir averages approximately 152 days [16]. Alum Creek Dam is located on Alum Creek, a tributary of Big Walnut Creek that drains into the Scioto River south of Columbus. The Hoover and Alum Creek Reservoirs are more characteristic of a lacustrine environment, resulting in a more classic reservoir system [16].

Water to these reservoirs is derived from their riverine watersheds north of Columbus, Ohio. The drainage area of Griggs-O’Shaughnessy is ~2700 km^2^, and Hoover’s is ~500 km^2^. Between 50–85% of the Griggs-O’Shaughnessy system (i.e., the Scioto River watershed) north of the reservoirs is agricultural, while 40–75% of the Alum Creek-Big Walnut watershed is cultivated. Both of these river systems lie in the Eastern Corn Belt Ecoregion, where the major crops are corn and soybean. The addition of fertilizer to the corn crops and the fixation of N_2_ by the legumes produce surface waters with high concentrations of nitrate. The nitrate concentrations in the river waters draining into the reservoirs are enhanced by the usage of tile drains within the croplands, which can rapidly transfer nitrate from soils into the river systems during rain and snow melt events, thereby bypassing recharge [17,18]. Concentrations of nitrate in the upstream, agricultural portion of the Scioto River have been measured as high as ~790 μM [19], and the Ohio EPA has described the upper Scioto to have moderate water quality issues [20]. Therefore, the primary source of water into these reservoirs has been impacted by agriculture, and the nitrate is derived from the upper drainages.

### 2.3. Collection, Storage, and Analyses of Water Samples

Event precipitation (*n* = 119) was collected using a precipitation collector located at the Byrd Polar Research Center on Ohio State’s campus (latitude: 40.0025; longitude: −83.0390). This collector consisted of a plastic funnel (top diameter = 248 mm; height = 319 mm), tygon tubing, and a 1000 mL Nalgene high density polyethylene (HDPE) bottle. Precipitation samples were collected in 20 mL plastic scintillation vials for anion analysis. Samples were then stored in the dark and chilled at 4 °C until time for analysis, usually within 1–3 months of collection.

Precipitation event volumes and daily temperatures were measured at Waterman Farm of The Ohio State University, which is <1 km north of the precipitation collector. These data are reported online through the Ohio Agricultural Research and Development Center [21].

Reservoir water collection was completed weekly at Griggs Reservoir (*n* = 31) due to its proximity to the university, and all other reservoirs were sampled on a monthly basis (May 2010–November 2011) by an individual wearing vinyl gloves (O’Shaughnessy *n* = 16, Hoover *n* = 17, and Alum *n* = 16). Water was collected at the farthest point on each reservoir pier, and reservoir samples were syringe filtered in the field using 0.4 µM Nucleopore filter caps into a 60 mL pre-cleaned Nalgene low density polyethylene (LDPE) bottle for anion analysis. Samples were refrigerated and stored in the dark until analysis.

Tap waters (*n* = 54) were collected from a residence tap in downtown Columbus supplied by the DRWP weekly over the study from May 2010 to November 2011. Water from the tap was allowed to flow for ~5 seconds, and an anion aliquot was collected and stored as the precipitation samples.

Major anion concentrations were analyzed using ion chromatography (Dionex DX-120) at Ohio State University, within 1–2 months of collection using the techniques of Welch et al. [22]. The anion precision calculated by relative standard deviation was Cl^−^ ≤ 4.4%, NO_3_^−^ ≤ 3.0% and SO_4_^2−^ ≤ 3.6%, respectively, of 100 measurements.

## 3. Results and Discussion

### 3.1. Meteorology

**Figure 2:**
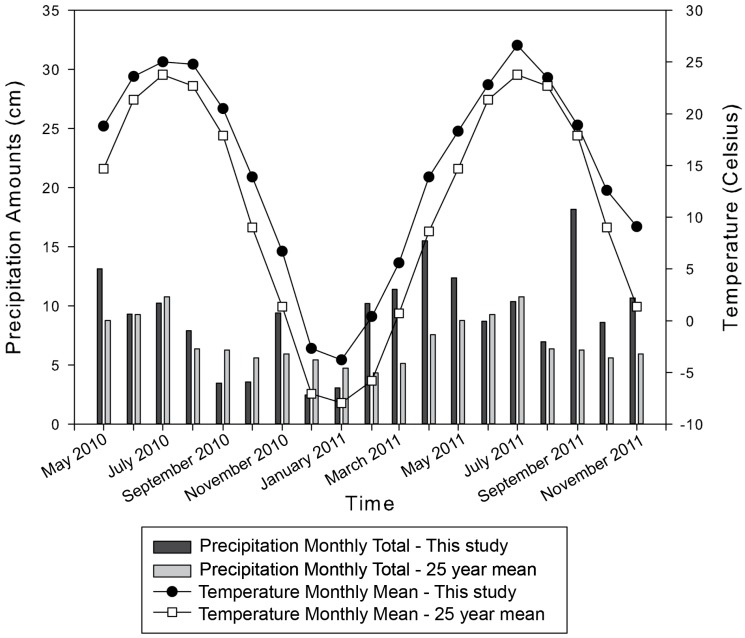
Monthly precipitation totals and average monthly air temperatures across the study during May 2010 to November 2011 in comparison to the 25 year monthly precipitation totals and average monthly air temperatures [13]. Data gathered from a weather station within the network of Ohio Agricultural Research and Development Center (OARDC) of The Ohio State University [21].

These meteorology data were previously published in Leslie et al. [13], and are noted here for context. The precipitation totals for this study were above the 25-year average monthly precipitation totals for central Ohio for more than half of the months in this study (May 2010 to November 2011; Figure 2). Monthly mean temperatures were slightly above normal compared to the 25-year monthly average temperature (Figure 2). The wettest year on record in Columbus was 2011 (total annual precipitation of 139.6 cm), with a previous record of 135.0 cm in 1990. Columbus also experienced the third wettest spring on record in 2011 (44.8 cm, with previous record of 48.8 cm in 1882), and the second wettest fall on record (38.1 cm, with the previous record of 39.4 cm in 1881) [23]. It is expected that these high rainfall periods had a very different impact on the reservoir/water system than other, more average precipitation years. Higher precipitation rates will also signify greater amounts of runoff, increasing concentrations of ions in these reservoirs as previously noted by Allen [16].

### 3.2. Precipitation Anion Chemistry

Cl^−^ concentrations were as high as 1.2 mM, with the average value only 0.03 mM. The days that the three highest Cl^−^ concentrations occurred were 30 December 2010, 18 January 2011, and 1 February 2011, when rain samples turned to ice prior to collection. These extremely high winter Cl^−^ values may be due, at least in part, to the nearby addition of road de-icing salt around The Ohio State University campus, and thus may be contaminated. However, we have included them in the data set. A statistical analysis using Pearson correlations was done using the day of year, precipitation amount, air temperature, Cl^−^, NO_3_^−^, and SO_4_^2−^ (Table 1). Significant relationships were noted if the variables had a *p*-value < 0.05. Cl^−^ concentrations in precipitation had a significant negative relationship with air temperature, and a significant positive correlation with NO_3_^−^ and SO_4_^2−^.

NO_3_^−^ concentrations were as high as 0.34 mM, which occurred on 8 April 2011, with an average NO_3_^−^ concentration of 0.04 mM. A significant negative correlation existed between NO_3_^−^ and precipitation amount, with a positive correlation between SO_4_^2−^ and Cl^−^.

SO_4_^2−^ concentrations ranged as high as 0.61 mM, which occurred on 23 October 2010, with an average concentration of 0.03 mM. A significant relationship was seen between SO_4_^2−^ and precipitation amount, Cl^−^, and NO_3_^−^. A negative correlation existed between precipitation amount and SO_4_^2−^. Sulfate concentrations were positively correlated with Cl^−^ and NO_3_^−^. These data were consistent with increased precipitation amounts decreasing, or diluting out, the anion concentrations.

### 3.3. Reservoir Water Anion Chemistry

The Pearson correlation coefficients of significance with a *p*-value <0.05 for reservoir data are presented in Table 2, Table 3, Table 4 and Table 5. All variables that were significantly correlated varied from reservoir to reservoir. Many similarities did occur between the previously mentioned connected/comparable systems of Griggs-O’Shaughnessy and Alum-Hoover. A significant positive correlation occurred between Cl^−^ and SO_4_^2−^ in both reservoir systems of flow-through and lacustrine.

As noted above, the Griggs-O’Shaughnessy (henceforth G-O) system functions more like a riverine system, while the Alum-Hoover (henceforth A-H) system is more like a lacustrine system [16]. This difference is reflected in most of the anion data. The G-O system is much more variable through time with a shorter residence time of 26 days and 5× the drainage area of A-H; signifying more runoff, less mixing, and acting as a plug flow reactor model (PFR) to control anion concentrations [16]. The A-H system has relatively constant concentrations with a longer residence time of 152 days and a smaller drainage area; resulting in more mixing with less runoff to describe a completely mixed flow reactor model (CMFR) [16].

**Figure 3:**
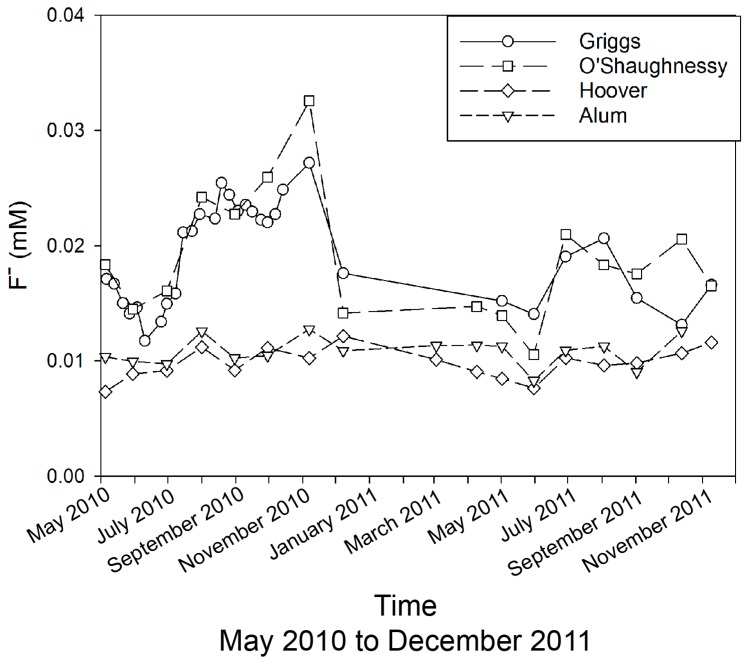
Fluoride concentrations in reservoirs (Griggs, O’Shaughnessy, Hoover, and Alum) across May 2010 to November 2011.

The F^−^ concentrations in the reservoir water are all below 34 µM, with G-O values being as much as 2× to 3× higher than the A-H values through most of the year (Figure 3). The G-O shows higher concentrations in the late summer/fall, with the highest concentrations in July–November of 2011 during times of higher precipitation. These F^−^ values, especially those of the A-H system, are within the range and close to the mean values of 13 µM and 16 µM, for South Asia and United Kingdom rivers, respectively [24,25], suggesting potential baseline concentrations for central Ohio. The reason for the higher concentrations in G-O is not known. It should be noted that there is a difference in headwater lithologies between G-O and A-H, where G-O is of limestone/dolomites and shale, while A-H consists primarily of shale. For example, the mineral fluorite (CaF_2_) is known to be associated with Silurian aged dolomites in Ohio [26].

**Figure 4:**
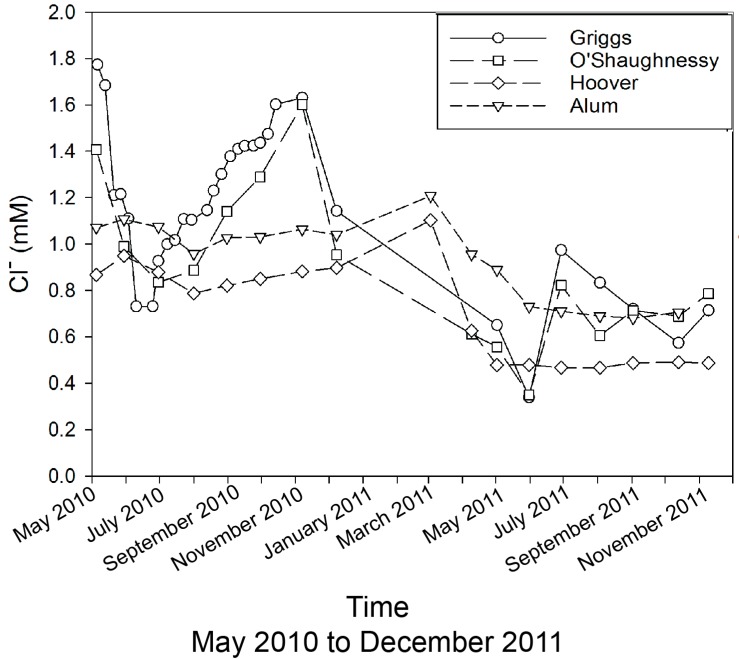
Chloride concentrations in reservoirs (Griggs, O’Shaughnessy, Hoover, and Alum) across May 2010 to November 2011.

The Cl^−^ concentrations in these reservoirs ranged from <0.1 to 1.8 µM, with generally slightly lower concentrations in the A-H system (Figure 4). The mean Cl^−^ value in “natural” river systems in North America has been determined to be ~0.2 mM, while the “actual” mean is 0.26 mM [27]. The majority of the reservoir values are above these concentrations, indicating above average input of Cl^−^ into these systems. This Cl^−^ comes from the addition of road de-icing salt [5,28]. However, the highest concentrations are in the G-O systems, rising through the summer into early winter, and in the late spring/early summer period. Although Gardner and Carey [28] have documented a continual flux of de-icing salt from major highways in Columbus throughout the year, it is unlikely that this is the primary source of Cl^−^ to these reservoirs in the summer months. The direct source of this Cl^−^ is unknown, but reservoir Cl^−^ systematics could reflect a dilution or concentration depending on precipitation amounts.

**Figure 5:**
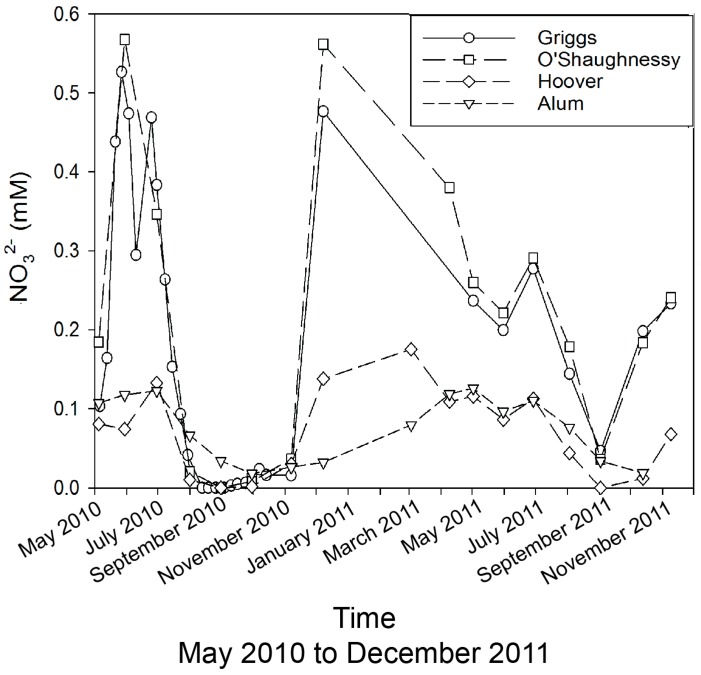
Nitrate concentrations in reservoirs (Griggs, O’Shaughnessy, Hoover, and Alum) across May 2010 to November 2011.

Nitrate concentrations in the A-H fluctuate around 0.1 mM, with lower concentrations in the late summer/early fall that we attribute to biological uptake within the watershed, possibly in the reservoirs themselves (Figure 5). The G-O data show similar decreases in the summer/fall to concentrations observed in A-H, but the G-O system has early summer and winter concentrations approaching 0.6 mM (Figure 5). The high values in the spring are probably due to runoff from recently fertilized agricultural lands in the northern part of the watershed, but it is unclear what the source of higher winter values are. During the winter increase in NO_3_^−^, both the Cl^−^ and SO_4_^2−^ values are decreasing, implying different sources of these solutes to these reservoirs. Previous work in central Ohio streams/rivers affected by agricultural activities has demonstrated increases as large as 90% from summer NO_3_^−^ lows to spring NO_3_^−^ highs [5]. Nitrate concentrations as high as 0.67 mM and values as low as 0.04 mM have recently been observed in agriculturally dominated streams in central Ohio [5]. This range of NO_3_^−^ values is similar to those observed by us in these reservoir waters.

**Figure 6:**
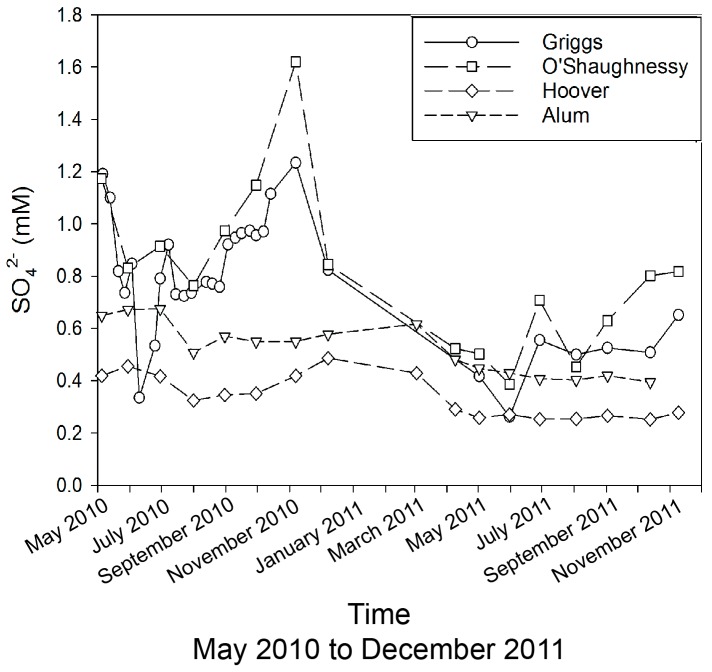
Sulfate concentrations in reservoirs (Griggs, O’Shaughnessy, Hoover, and Alum) across May 2010 to November 2011.

Sulfate concentrations show a similar pattern to Cl^−^. In general, the G-O system has higher concentrations with ~2-fold variations, while the A-H system has lower concentrations (0.3–0.6 mM) (Figure 6). The average North American river SO_4_^2−^ concentration is 0.19 mM, so even the A-H waters have relatively elevated values, while the highest G-O values of ~1.6 mM are close to an order of magnitude above the North American mean. The G-O SO_4_^2−^ time series closely resembles the Cl^−^ pattern, perhaps suggesting similar sources or similar processes controlling their distributions.

Both the Griggs-O’Shaughnessy and Hoover-Alum followed similar trends in anion concentrations over time. Chloride and sulfate concentrations were higher in May 2010, decreased during June 2010, and increased steadily until November 2010. This was followed by a steady decrease in concentrations through June 2011. Concentrations rose in July 2011, and then Cl^−^ decreased, while SO_4_^2−^ remained relatively constant through the rest of the time series. Nitrate increased in June 2010 and then decreased corresponding with decreases in Cl^−^ and SO_4_^2−^. Low NO_3_^−^ concentrations occurred through November 2010, with a spike in December 2010. Then NO_3_^−^ values steadily decreased until September 2011, with a minor increase into October–November 2011. Hoover and Alum Creek Reservoirs had relatively constant concentrations of SO_4_^2−^ and NO_3_^−^ throughout the study with minimal fluctuation of SO_4_^2−^ concentrations of <0.1 mM and with NO_3_^−^ concentrations fairly constant. The concentrations of Cl^-^ in Alum Creek Reservoir changed by 0.3 mM throughout the time period, and Cl^−^ reached its peak of ~1.2 mM in March 2011. The Cl^−^ concentrations continually decrease through November 2011. In the Hoover Reservoir, Cl^−^ values remained constant until December 2010 and increased by March 2011. Hoover Cl^−^ concentrations decreased to their lowest level in July 2011, and by August 2011, concentrations returned to the previous Cl^−^ concentration observed in June 2011.

Reservoir management and water supply dynamics are important in order to anticipate water resources availability [29]. Higher anion concentrations could signify lower reservoir volumes through evapoconcentration and/or water consumption. Precipitation amounts were near the 25-year average during this period in 2010. Reservoir waters of lower anion concentrations occurred during June–November 2011, which likely indicates that higher precipitation volumes diluted reservoir anion concentrations. We suggest that the observed reservoir behavior that occurred in 2010 was due to evaporation, while in 2011, increased precipitation amounts diluted anion concentrations. Normally, conservative elements such as Cl^−^ can be utilized to help determine evaporation effects, but the high Cl^−^ concentrations in these reservoir waters and the lack of knowledge about its sources make this more challenging. This aspect of the systems’ anion geochemistry complicates discerning between reservoir evaporative loss and variations in precipitation sources.

### 3.4. Residential Tap Waters

Over the time of this investigation, Cl^−^ and SO_4_^2−^ followed similar trends, while NO_3_^−^ and F^−^ varied together as well. Chloride concentrations increased to 6.9 mM on 6 February 2011 with an average concentration of 1.5 mM. The highest SO_4_^2−^ concentration of 1.8 mM occurred on 24 October 2010 with an average concentration of 1.2 mM. Fluoride and nitrate concentrations were minimal, <0.05 mM, with higher nitrate levels of ~0.05 mM occurring in June 2010, December 2010, and January 2011.

Pearson correlations were calculated for the tap water samples (Table 6). Significant positive correlations existed between day of year–SO_4_^2−^, F^−^–Cl^−^, F^−^–NO_3_^−^, F^−^–SO_4_^2−^, Cl^−^–NO_3_^−^, and Cl^−^–SO_4_^2−^. Significant negative relationships occurred between day of year–Cl^−^, day of year–NO_3_^−^, temperature–F^−^, temperature–Cl^−^, temperature–NO_3_^−^, and temperature–SO_4_^2−^. Chloride and nitrate had lower concentrations later in the calendar year, while SO_4_^2−^ had higher concentrations later in the year. The negative correlations with temperature indicate that as the air temperature increases, solute concentrations decrease. Solute–solute correlations of F^−^ and Cl^−^ with NO_3_^−^, SO_4_^2−^, and Cl^−^ have positive correlations, demonstrating a co-variance.

### 3.5. Anion Comparison of Precipitation to Reservoir To Tap

**Figure 7:**
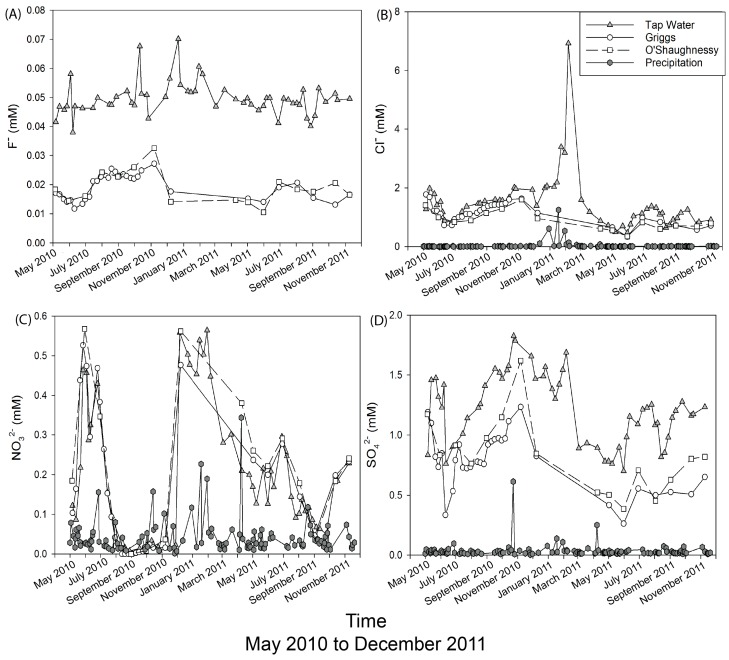
Anion concentrations in the tap water, Griggs Reservoir, O’Shaughnessy Reservoir, and precipitation across May 2010 to November 2011, with each graph representing a different anion (**A**) fluoride (**B**) chloride (**C**) nitrate (**D**) sulfate.

Plots to compare over time the four anionic species from each of the three water types (precipitation ➔ reservoir ➔ tap) are shown in Figure 7. The F^−^, Cl^−^, NO_3_^−^, and SO_4_^2−^ concentrations in the precipitation were all equal or lower than in the reservoirs with a few exceptions. Seemingly anomalously high NO_3_^−^ concentrations in precipitation collected in April 2011 are thought to be due to a local effect near the collection site. In general, the tap water has concentrations similar or lower than the reservoir. Not surprisingly, the tap has in most cases 2× higher F^−^ than the reservoir water, as F^−^ is being added as an anti-tooth decay agent during the treatment process. Our highest observed F^−^ value is very similar to that reported by the City of Columbus for tap water (~62 µM) [30]. At most times, the SO_4_^2−^ concentration in the tap water were greater than the reservoir value. This should be expected, given the fact that alum (Al-SO_4_ salts) is used to treat the water before distribution. In most cases, the Cl^−^ in the tap water was higher than the reservoirs (Figure 7B), but this difference is rather small (~0.1–0.2 mM). The rather large difference in January to March 2011 may be because of an extensive ice cover on the reservoirs themselves. The reservoirs were not sampled during this time, while the tap water was. Here, the tap water may well represent the reservoir Cl^−^ concentrations due to the influx for Cl^−^ from the local highway de-icing and potentially other anthropogenic activities, but we have no reservoir water to substantiate this hypothesis.

## 4. Conclusions

This work describes an anionic characterization of precipitation, surface reservoir waters, and residential tap waters to better understand the relationship of the hydrologic residence time on the tap water chemistry, as well as seasonal variations. The highest Cl^−^ tap water concentration occurred in early February 2011, while increases in NO_3_^−^ occurred in both early summer and the middle of winter. Tap water Cl^−^ and NO_3_^−^ concentrations did not exceed the EPA drinking water standard concentrations. The tap water had concentrations similar to or lower than the reservoir water. Tap water had higher F^−^ and SO_4_^2−^ concentrations, suggesting that they are added during the treatment of the reservoir water prior to residential distribution. In general, the anion concentrations in the precipitation are all equal to or lower than the reservoir waters. The input of these anions from the interaction of precipitation and the watershed soils greatly affect the concentrations of these constituents. This is particularly the case for nitrate, as agricultural practices far afield from the reservoirs themselves are the most influential. While 2011 proved to be the wettest year on record in Columbus, Ohio, one implication of this was a shorter residence time of reservoir water to the residential tap. 

## Figures and Tables

**Table 1:** Pearson correlation coefficients of significance for May 2010 to November 2011 precipitation data relationships with a *p*-value of <0.05.

2010–2011 Precipitation						
*n* = 119	Day of Year	Amount	Temp	Cl^−^	NO_3_^−^	SO_4_^2−^
Day of Year	1					
Amount		1				
Temp	0.18		1			
Cl^−^			−0.25	1		
NO_3_^−^		−0.32		0.46	1	
SO_4_^2−^		−0.20		0.68	0.46	1

**Table 2:** Pearson correlation coefficients of significance for Griggs Reservoir data relationships with a *p*-value < 0.05.

Griggs Reservoir					
*n* = 31	Day of Year	Temp	Cl^−^	NO_3_^−^	SO_4_^2−^
Day of Year	1				
Temp	−0.47	1			
Cl^−^			1		
NO_3_^−^	−0.42		−0.41	1	
SO_4_^2−^			0.93		1

**Table 3:** Pearson correlation coefficients of significance for O’Shaughnessy Reservoir data relationships with a *p*-value < 0.05.

O’Shaughnessy Reservoir					
*n* = 16	Day of Year	Temp	Cl^−^	NO_3_^−^	SO_4_^2−^
Day of Year	1				
Temp		1			
Cl^−^	0.54		1		
NO_3_^−^				1	
SO_4_^2−^	0.59		0.96		1

**Table 4:** Pearson correlation coefficients of significance for Hoover Reservoir data relationships with a *p*-value < 0.05.

Hoover Reservoir					
*n* = 17	Day of Year	Temp	Cl^−^	NO_3_^−^	SO_4_^2−^
Day of Year	1				
Temp		1			
Cl^−^			1		
NO_3_^−^				1	
SO_4_^2−^		−0.56	0.91		1

**Table 5:** Pearson correlation coefficients of significance for Alum Creek Reservoir data relationships with a *p*-value < 0.05.

Alum Creek Reservoir					
*n* = 16	Day of Year	Temp	Cl^−^	NO_3_^−^	SO_4_^2−^
Day of Year	1				
Temp		1			
Cl^−^		−0.55	1		
NO_3_^−^	0.81			1	
SO_4_^2−^			0.92		1

**Table 6:** Pearson correlation coefficients of significance for residential tap water, distributed from Dublin Road Water Plant, data relationships with a *p*-value of <0.05.

Dublin Road Water Plant Tap Water						
*n* = 54	Day of Year	Temp	F^−^	Cl^−^	NO_3_^−^	SO_4_^2−^
Day of Year	1					
Temp		1				
F^−^		−0.31	1			
Cl^−^	−0.28	−0.51	0.35	1		
NO_3_^−^	−0.43	−0.61	0.35	0.36	1	
SO_4_^2−^	0.33	−0.35	0.29	0.61		1
